# Effect of manual decongestive therapy on cardiac preload in critically ill patients: a randomized controlled trial

**DOI:** 10.1186/s13613-025-01453-z

**Published:** 2025-03-24

**Authors:** Matthias J. Posch, Christian I. Schwer, Johannes Kalbhenn, Joachim Bansbach

**Affiliations:** Department of Anesthesiology and Critical Care, Faculty of Medicine, Medical Center – University of Freiburg, University of Freiburg, Freiburg I.Br., Germany

**Keywords:** Manual decongestive therapy, Lymphatic drainage, Cardiac preload, Critical care, Fluid balance, Capillary leak

## Abstract

**Background:**

Capillary leakage is common in critical illness and can lead to intravascular hypovolemia and edema. Fluid balance, however, is crucial to optimize cardiac preload, vascular filling and tissue perfusion. Intravenously administered fluids are rapidly distributed to the extravascular spaces and further increase edema with consecutive harm for impeded wound healing, weakness, distribution of pharmaceutics to the third space and patient discomfort. We hypothesized that manual decongestive therapy (MDT) followed by elastic bandaging increases cardiac preload and reduces interstitial edema and thus, offers a promising approach to restore the imbalance in fluid distribution between the interstitium and the intravascular space in critically ill patients.

**Methods:**

From November 2021 to May 2023, 34 critical ill requiring advanced hemodynamic monitoring with thermodilution-calibrated pulse contour analysis were randomized to either standard care or MDT followed by elastic bandaging for 24 h. Global end-diastolic volume index (GEDI) as a marker of the cardiac preload was measured 15, 30, 60 min and 24 h after MDT. Wrist and ankle circumferences were measured as markers of the extent of local interstitial edema.

**Results:**

In the intervention group, a significant increase in Δ GEDI was observed 15 min [median 48 (IQR 82) to median −19 (IQR 39)], 60 min [median 75 (IQR 106.5) to median −11 (IQR80)] and 24 h [median 107 (IQR 153) to median −16 (IQR 114)] after the study intervention compared to the control group.

After 24 h ankle [median 23.5 (IQR 5) cm to median 24 (IQR 6) cm, p < 0.0001] and wrist] median 18 (IQR 2) cm to median 19 (IQR 2) cm, p < 0.0001] circumferences were increased significantly in the control group. In the intervention group a significant reduction in the ankle circumference [median 24.5 (IQR 5) cm to median 24 (IQR 4.5) cm, p < 0.0001] and a significant reduction in the wrist circumference [median 20 (IQR 3.8) cm to median 18 (IQR 3.5) cm, p < 0.0001], was observed after 24 h.

**Conclusions:**

MDT increases cardiac preload and helps to reduce interstitial fluid overload and edema in critically ill patients.

*Trial registration:* This prospective randomized controlled trial was registered at the German Clinical Trials Register DRKS00026226 on 17/09/2021.

**Graphical Abstract:**

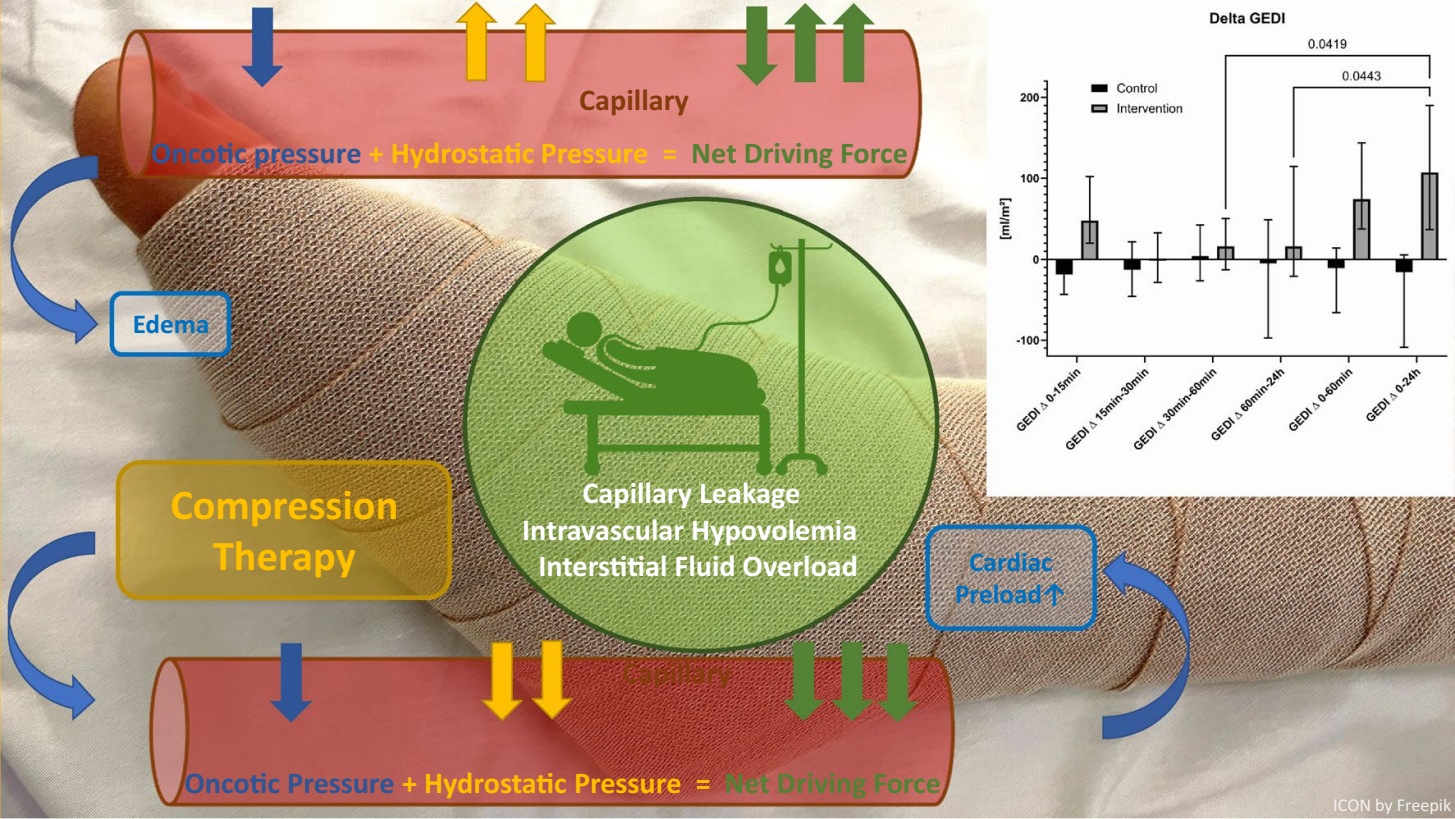

## Background

Almost every patient receives intravenous fluids while treated in intensive care to achieve adequate organ perfusion and tissue oxygenation [1, 2]. While dehydration and hypovolemia can worsen renal and other organ function, excessive fluid therapy can be the cause of edema and fluid overload, which are associated with increased mortality [3, 4].

Fluid resuscitation and impaired endothelial glycocalix lead to fluid overload with organ dysfunction and edema in many critically ill patients, a state that has been described as fluid accumulation syndrome or capillary leak syndrome, respectively [5, 6]. Next to capillary permeability and the osmotic pressure gradient, the level of hydrostatic pressure is another important and potentially modifiable factor not to be neglected in this context [7, 8].

Manual decongestive therapy (MDT) is a well-established physiotherapeutic method for reducing edema of various etiologies by applying skin care, manual lymphatic drainage and compression with multilayered bandage [9]. It aims not only to inhibit further fluid transfer from the vasculature into the interstitium, but also to reduce maldistribution of body fluid [10–12]. In critically ill patients, it may therefore not only reduce edema but also improve cardiac preload without additional fluid resuscitation and indeed, compression bandaging has been shown to reduce fluid requirements in critically ill patients before [13].

Global end-diastolic volume (GEDV) is the hypothetic volume of blood contained assuming the four chambers of the heart being at the end of the diastole and a good indicator of cardiac preload in critically ill patients [13]. It can be measured using the transpulmonary thermodilution technique and is commonly expressed in relation to the body surface area as the global end-diastolic volume index (GEDI) [14–16].

We hypothesized that MDT followed by elastic bandaging increases the GEDI in critically ill patients and therefore performed this prospective, single-center, open-label, randomized, and controlled trial.

## Material and methods

### Study design

This prospective, single-center, randomized, open-label clinical trial was conducted at the anesthesiologic intensive care unit of the University Medical Center, Freiburg, Germany. The study protocol was approved by the Ethics Committee of the University Medical Center Freiburg (EK 21-1256) and was registered at the German Trials Register (DRKS00026226). Enrolment took place between November 2021 and May 2023.

Twice 17 identical-looking opaque envelopes containing cards labeled with either “intervention” or “no intervention” were prepared, sealed and shuffled before the start of the study. All patients admitted to the intensive care unit were assessed for eligibility. Written informed consent was obtained from patients or their legal representatives. Patients were then randomized to one of two groups by drawing one of the envelopes. The intervention group received standard care and MDT, while the control group received standard care without MDT.

### Inclusion and exclusion criteria

Inclusion criteria included the presence of edema as determined by the supervising study physician and an indication for off-study thermodilution-calibrated pulse contour analysis. Exclusion criteria included lack of consent from the patient or their legal representative, extreme hemodynamic instability defined as a change in cumulative dose of epinephrine or norepinephrine greater than 0.15 µg/kg/min within the 12 h prior to enrollment, and any contraindication to manual MDT according to the treating physician. Patients under 18 years of age and pregnant women were also excluded.

### Measurements and experimental protocol

After at least 10 min in the supine position, i.e. bed in 0° flat position with 30° upper body elevation, hemodynamic measurements were started in both groups to determine baseline characteristics. Calibration of the thermodilution-calibrated pulse contour analysis system (PiCCO₂ PC8500 Pulsion Medical Systems, Feldkirchen, Germany) was performed according to its instruction manual at all specified measurement times. GEDI and other hemodynamic parameters such as intrathoracic blood volume index (ITBVI), extravascular lung water index (EVLWI), cardiac output, heart rate, systolic, mean and diastolic arterial blood pressure, central venous pressure and urine output were digitally recorded. The circumferences of the extremities to be treated with MDT were measured, documented and the measurement points were marked.

### Intervention

**Fig. 1:**
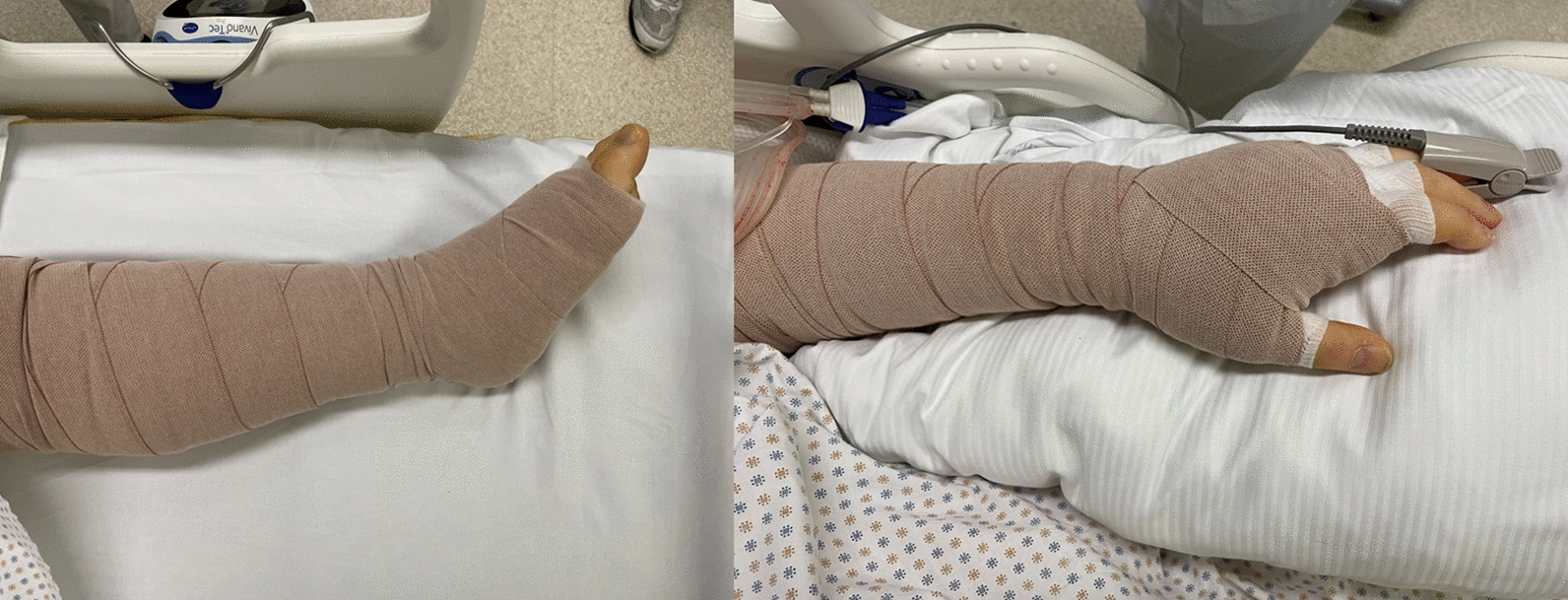
Manual decongestive therapy of the upper and lower limb

MDT was performed on all four extremities by a qualified physiotherapist in accordance with available guidelines [17]. The first phase involved skin care and targeted light manual drainage, followed immediately by multi-layer bandaging as a method of continuous lymphatic drainage. Bandaging was performed with Cellona^®^ synthetic underpadding and Comprilan^®^ 5 m × 8 cm elastic short-stretch bandages (Fig. 1).

### End points

Primary endpoint:

The primary endpoint was GEDI 15, 30, 60 min and 24 h after MDT.

Secondary endpoints:

The secondary endpoints were limb circumference before and 24 h after MDT and changes in ITBVI, EVLWI, cardiac output, heart rate, systolic, mean and diastolic arterial blood pressure, central venous pressure and urine output before and 15, 30, 60 min and 24 h after MDT.

### Vasopressors and inotropes

We used the Vasoactive Inotrope Score (VIS) to visualise vasopressor and inotrope dosing and improve comparability between study groups [18].

### Data collection and statistics

To calculate the number of cases, GEDI was retrospectively compared in five patients with and without intervention before and 1 h after the study intervention. The mean of the differences in GEDI before and after the intervention was 89 ml/m^2^ with a standard deviation of ± 40 in the intervention group and −11.8 ml/m^2^ with a standard deviation of 40.3 in the control group. For sample size calculation, a change in GEDI of 80 ml/m2 was considered relevant. A standard deviation of 60 was conservatively assumed for both groups. A non-parametric two-sided Wilcoxon test was used to account for possible upward outliers. Since a difference between the two groups of 80 ml/m^2^ on average should be detected at a significance level of 5% with 90% power, 17 patients per group were required (Module MTT1, nQuery version 8.6.1.0). The Wilcoxon signed rank test was used in the primary confirmatory analysis.

Data were collected over a period of 13 months in modified Excel spreadsheets (Microsoft Excel 2016, Microsoft Corporation, Redmond, Washington, USA). Statistical analysis was performed using IBM SPSS Statistics (version 29.0.0.0, IBM Corporation, Armonk, New York, USA) and GraphPadPrism (version 9.5.1., GraphPad Software LLC/Insight Partners, New York, USA). Metric data were tested for normal distribution. If the P value was > 0.05, the null hypothesis was retained and the data was considered to be normally distributed. In this case, the differences of the means between the groups were calculated using the unpaired T-test and the standard deviation was specified. Otherwise, medians and the IQR were calculated and the “median test for independent samples” was used to calculate the differences. For calculation of differences between nominal data the Chi-square-test was used. Analyses between time points were performed using two-way ANOVA followed by multiple comparisons. A p-value of less than 0.05 was considered statistically significant. Graphs were generated using GraphPadPrism.

## Results

### Study cohort and baseline characteristics

Between November 2021 and May 2023 all patients admitted to ICU were screened. 36 patients fulfilled all inclusion criteria. Two patients refused to participate in the study.

A total of 34 patients were enrolled during the study period, 17 in the control group, and 17 in the intervention group. Patient characteristics did not differ between the study groups (Table 1). Demographic and hemodynamic parameters measured immediately before the start of the study intervention (Table 1) and hemodynamic and fluid status during the study period (Table 2) showed no statistically significant difference between the two study groups. No skin tears, cardiac decompensation or other adverse effects of MDT were observed in patients in the intervention group.

**Table 1:** Demographic and haemodynamic details at baseline

	Control (n = 17)	Intervention (n = 17)	P value
Demographics			
Male sex, n (%)	12 (71)	11 (65)	0.901
Age, years, mean (SD)	54.4 ± 17.17	58.6 ± 17.23	0.48
BMI (kg/m^2^) mean (SD)	28.7 ± 7.31	28.2 ± 6.53	0.84
Admission diagnosis			
Septic shock, n (%)	11 (64.7)	10 (58.8)	0.73
Haemorrhagic shock, n (%)	4 (23.5)	2 (11.8)	0.37
Lung transplant, n (%)	1 (5.9)	4 (23.5)	0.15
ARDS, n (%)	1 (5.9)	1 (5.9)	1
Status at inclusion			
SOFA-Score, mean (SD)	7.1 ± 3.1	7.8 ± 3.0	0.54
Mechanically ventilated, n (%)	10 (59)	11 (65)	0.72
Ventilation mode			
HFNOT, n (%)	7 (41.2)	6 (35.3)	0.72
CPAP + ASB	9 (52.9)	10 (58.8)	0.73
SIMV	1 (5.9)	1 (5.9)	1
PEEP (mbar) median (IQR)	10 (4.3)	10 (3)	0.66
Sedation, n (%)	2 (11.8)	1 (5.9)	0.55
RASS, median (IQR)	−2.5 (3)	−1 (5)	0.46
VIS, median (IQR)	3 (23.5)	6.5 (23)	0.49
ARF, n (%)	8 (47)	9 (53)	0.73
Hemodialysis, n (%)	0	1 (5.9)	0.94
ICU-days until intervention, median (IQR)	6 (4)	5 (4)	0.73
Hemodynamic parameters			
Heart rate [1/min], mean (SD)	95 (17)	94 (21)	0.436
Mean aterial pressure [mmHg], mean (SD)	80 (13)	76 (13)	0.18
Central venous pressure [mmHg], mean (SD)	12 (4.3)	14 (4)	0.112
SVRI [mmHg/l/min/m^2^], median (IQR)	1453 (683)	1427 (558)	1.0
PVPI, median (IQR)	2 (0.8)	2.1 (1.0)	1.0
GEDI [ml/m^2^], mean (SD)	726 (151)	669 (100)	0.095
GEF [%], mean (SD)	25 (7.23)	26 (8.54 >)	0.36
EVLWI [ml/kg/m^2^], median (IQR)	10 (4)	9 (5)	1.0
Cardiac Index [l/min/m^2^], mean (SD)	3.76 (1.12)	3.65 (0.97)	0.38

*RASS* Richmond Agitation and Sedation Scale, *HFNOT* High Flow Nasal Oxygen Therapy, *CPAP* + *ASB* Continuous Positive Airway Pressure + Assisted Spontaneous Breathing, *SIMV* Synchronized Intermittent Mandatory Ventilation, *PEEP* Positive Endexspiratory Pressure, *ARF* Acute Renal Failure, *ICU* Intensive Care Unit, *VIS* Vasoactive Inotropic Score, *SD* Standard Deviaton, *IQR* Interquartile Range, *SVRI* Systemic Vascular Resistance Index, *PVPI* Pulmonary Vascular Permeability Index, *GEDI* Global End-Diastolic Volume Index, *GEF* Global Ejection Fraction, *EVLWI* Extravascular Lung Water Index

**Table 2:** Time course of hemodynamics, vasopressor and inotropic requirements, fluid intake and fluid balance

		Baseline	15 min	30 min	60 min	24 h
Heart rate [1/min], median (IQR)	Control Intervention P	96 (21) 98 (289 1.0	94 (17) 100 (26) 1.0	91 (23) 100 (31) 0.73	90 (22) 97 (26) 0.73	98 (25) 95 (21) 1.0
Cardiac Index [l/m^2^], median (IQR)	Control Intervention P	3.5 (2) 3.6 (1.1) 0.49	4 (1.4) 3.8 (0.95) 1.0	3.6 (1.3) 3.9 (0.8) 0.27	3.5 (1.1) 4.4 (1.6) 0.17	4.1 (1.5) 4.1 (1.2) 0.73
Mean arterial Pressure [mmHg], median (IQR)	Control Intervention P	80 (14) 74 (18) 0.49	79 (21) 79 /27) 0.73	80 (13) 76 (23) 1.0	74 (14) 77 (14) 1.0	76 (21) 86 (29) 0.17
Central venous pressure [mmHg], median (IQR)	Control Intervention P	11 (6) 13 (5) 0.49	11 (6) 12 (9) 0.73	14 (8) 14 (10) 1.0	13 (9) 14 (6) 1.0	13 (12) 14 (10) 1.0
VIS, median (IQR)	Control Intervention P	6.7 (23.1) 4.0 (13.2) 0.49	6.7 (23.5) 1.26 (20.7) 0.49	6.7 (23.1) 2.0 (22.7) 0.49	9.0 (23.6) 1.41 (23.9) 0.49	6.7 (23.1) 4.0 (13.2) 0.49
Fluid intake since randomization [ml], median (IQR)	Control Intervention P	0 0			115 (135) 112 (56) 0.73	3404 (3934) 4095 (4095) 1.0
Diuresis [ml/h]? median (IQR)	Control Intervention P	0 0			101.7 (96.5) 89.8 (121.2) 0.75	1130 (2425) 1500 (2280) 0.73
Fluid balance [ml], median (IQR)	Control Intervention P	0 0			28 (162) 90 (243) 0.49	1284 (2899) 127 (2692) 0.49

### Primary endpoint

**Fig. 2:**
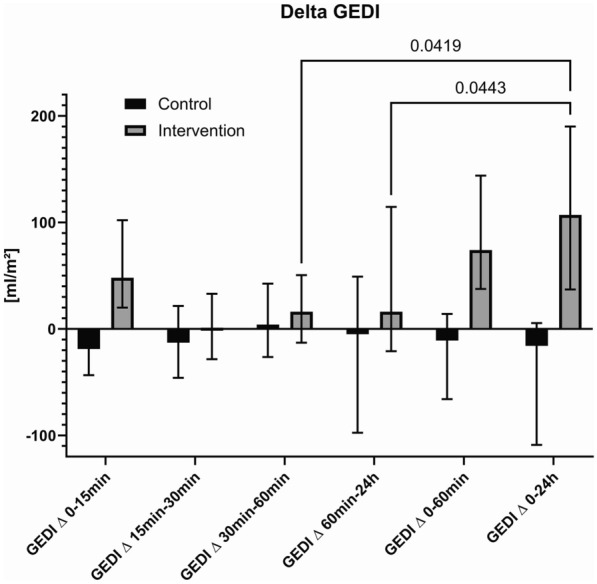
Comparison of Δ GEDI over time between control and intervention groups

In the intervention group, a significant increase in Δ GEDI was observed 15 min [median 48 (IQR 82) to median −19 (IQR 39)], 60 min [median 75 (IQR 106.5) to median −11 (IQR80)] and 24 h [median 107 (IQR 153) to median −16 (IQR 114)] after the study intervention compared to the control group (Fig. 2 and Table 3).

**Table 3:** Delta GEDI

		GEDI ∆15 min→ baseline	GEDI ∆30 min→ 15 min	GEDI ∆60 min→ 30 min	GEDI ∆60 min→ baseline	GEDI ∆24h→60min	GEDI ∆24 h→ 0 min
GEDI ∆, median (IQR)	Control Intervention P	−19 (39) 48 (82) 0.001	−13 (82) 1 (61.5) 0.73	4 (69) 16 (63.5) 0.17	−11 (80) 75 (106,5) < 0.001	−5 (146) 16 (165) 0.17	−16 (114) 107 (153) 0.001

### Secondary endpoints

In the intervention group, a significant reduction in the ankle circumference median 24.5 (IQR 5) cm to median 24 (IQR 4.5) cm, p < 0.0001) and a significant reduction in wrist circumference (median 20 (IQR 3.8) cm to median 18 (IQR 3.5) cm, p < 0.0001), was observed after 24 h.

**Fig. 3:**
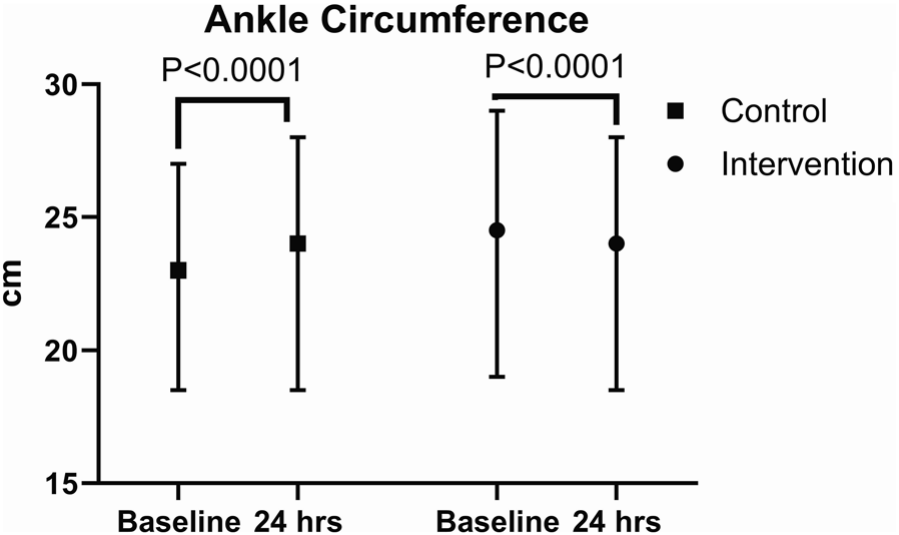
Ankle circumference at the beginning and end of the intervention compared with the control group

**Fig. 4:**
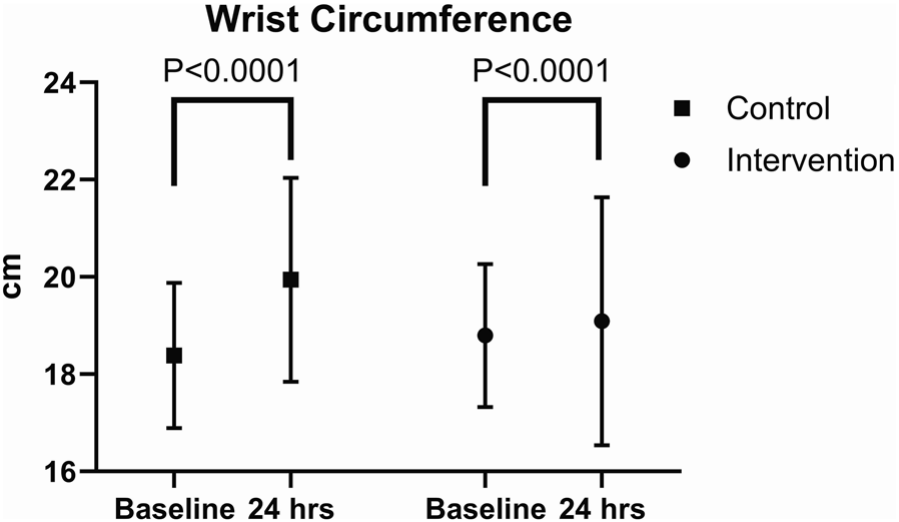
Wrist circumference at the beginning and end of the intervention compared with the control group

In the control group, ankle circumferences (median 23.5 (IQR 5) cm to median 24 (IQR 6) cm, p < 0.0001) and wrist (median 18 (IQR 2) cm to median 19 (IQR 2) cm, p < 0.0001) were increased significantly after 24 h (Table 4, Figs. 3, 4).

**Table 4:** Circumference of the wrist and ankle before and 24 h after the intervention

	Before	After	P-value
Circumference wrist [cm], median (IQR)			
Control	18 (2)	19 (2)	< 0.0001
Intervention	20 (3.8)	18 (3.5)	< 0.0001
Circumference ankle [cm], median (IQR)			
Control	23.5 (5)	24 (6)	< 0.0001
Intervention	24.5 (5)	24 (4.5)	< 0.0001

Analysis of the other secondary endpoints ITBVI, EVLWI, heart rate, systolic, mean and diastolic arterial blood pressure, central venous pressure, urine output and fluid balance showed no statistically significant differences between the control and intervention group at any of the defined time points.

There were no significant differences between the two study groups in the administration of fluids at 1 h and 24 h after the study intervention. Only crystalloid fluids (i.v.) and water (p.o.) were used (Table 2).

## Discussion

This prospective, randomized clinical trial provides evidence that MDT followed by elastic bandaging temporarily increases cardiac preload and significantly reduces ankle circumference in critically ill patients.

In the critically ill patients among other factors capillary leak lead to interstitial fluid accumulation with adverse effects on clinically relevant outcomes [5, 6]. However, the effect of loop diuretics on mortality in adult intensive care patients with fluid overload is uncertain [19] and, despite the excess of interstitial fluid, patients might experience intravascular hypovolemia with concomitant hemodynamic instability [3, 6, 20]. In such patients fluid resuscitation has no lasting effect and is even detrimental as it worsens the interstititial fluid overload not only by reduction of the oncotic pressure but also by further disrupting the physiological endothelial barrier [21–23]. In this context MDT could provide optimization of cardiac preload by mobilizing interstitial fluid without the need for additional fluid administration, but with the benefit of reducing potentially harmful tissue edema.

While the efficacy of MDT as a method of treating lymphedema has been well characterized [10–12], the direct hemodynamic effects of compression techniques in critically ill patients have not been evaluated. Nevertheless, an enhancement in cardiac output was documented in patients who were assessed to ascertain the safety of compression techniques in individuals with chronic heart failure [24]. In our patients, cardiac preload was significantly increased after 15 and 60 min after initiation of MDT. Early increase in GEDI could have been confounded by the provoked adrenergic activation of the procedure itself yet, this seems rather unlikely after 60 min. Besides there was no significant increase in heart rate nor blood pressure, which would have been expected in the case of adrenergic activation.

MDT is an effective method to redistribute body fluid and reduce edema [10]. The effect is presumably based on different mechanisms. By applying pressure to edematous tissue, MDT may inhibit further fluid transfer from the capillaries to the interstitium and lymphatic drainage will mobilize fluid via the lymphatic system to the vasculature [10–12]. Another important effect of MDT might be the redistribution inside the vascular system. Up 70% of the circulating blood is thought to be part of the unstressed volume [25], which in contrast to the stressed volume is not creating vascular pressure [26]. As the amount of unstressed volume is highly dependent on the surrounding tissue pressure and the venomotor tone, this may be another important mechanism for the potential of MDT to optimize the cardiac preload [27].

In our cohort, however, the increases in GEDI was no longer significant after 24 h. Possible explanations are, that the redistribution of fluid is similar to the volatility of a crystalloid fluid bolus in critically ill patients [22], and that the compression effect of the dressing decreases over time and therefore may have been too small to be detected with the selected number of cases. This seems all the more likely as ΔGEDI to baseline in the patients receiving MDT was significantly higher after 24 h compared to patients, who received standard care. It is important to consider that when MDT is applied to critically ill patients, the provoked fluid shift may also lead to unwanted fluid accumulation in other parts and organ systems [5]. Indeed, venous congestion may further alter renal function in patients with compromised circulation, which could also be an explanation that the induced increase of cardiac preload did not affect diuresis in our patients [28, 29]. However, we did not observe any changes of EVLWI during MDT, which strongly suggests that MDT did not result in a relevant pulmonary congestion in our cohort. These observations align with the findings of a study that investigated the influence of compression bandaging in patients at the onset of septic shock. The study even observed an improvement of organ function [13]. Another important finding is of our study is, that bandage compression does not necessarily cause adverse side effects such as skin ulcers and bacterial or fungal infections, contrary to previous reports in the literature [10, 13]. The relatively low complication rate may be explained by the limited use of MDT to the extremities, the limited duration of 24 h and the performance by highly experienced, specially trained and certified physiotherapists.

Our study has a number of limitations. First, this was a single-center study with a small number of heterogeneous patients, so the conclusions cannot be generalized. Secondly, blinding was not possible as the MDT and the bandaging were obvious to the physician calibrating and measuring the thermodilution system.

Thirdly, the follow-up time was limited to 24 h, and therefore our data cannot provide information on whether MDT might affect long-term fluid balance and other subsequent complications influencing the outcome of critically ill patients. Further studies with a multicenter design and longer follow-up are needed to confirm our findings and to elucidate whether consistent use of MDT can help to reduce fluid overload and to improve the outcome of critically ill patients.

## Conclusion

MDT represents a straightforward, non-invasive, and secure approach to augment the cardiac preload without further fluid administration, while concurrently attenuating tissue edema in patients who are critically ill. To fully actualize the potential of this promising method for the management of fluid overload in critically ill patients, it is imperative to conduct additional randomized trials.

## Data Availability

The data sets used and analyzed during the current study are available from the corresponding author upon reasonable request.

## Abbreviations

MDT: Manual decongestive therapy
GEDV: Global end diastolic volume
GEDI: Global end diastolic volume index
ITBVI: Intrathoracic blood volume index
EVLWI: Extravascular lung water index
SD: Standard deviation
CI: Confidence interval

## References

[1] Finfer S, Myburgh J, Bellomo R. Intravenous fluid therapy in critically ill adults. Nat Rev Nephrol. 2018;14:541–57. 30072710 10.1038/s41581-018-0044-0

[2] Vincent J-L, De Backer D. Circulatory shock. N Engl J Med. 2013;369:1726–34. 24171518 10.1056/NEJMra1208943

[3] Messmer AS, Zingg C, Müller M, Gerber JL, Schefold JC, Pfortmueller CA. Fluid overload and mortality in adult critical care patients—a systematic review and meta-analysis of observational studies*. Crit Care Med. 2020;48:1862–70. 33009098 10.1097/CCM.0000000000004617

[4] Payen D, de Pont AC, Sakr Y, Spies C, Reinhart K, Vincent JL, et al. A positive fluid balance is associated with a worse outcome in patients with acute renal failure. Crit Care. 2008;12:R74. 18533029 10.1186/cc6916 PMC2481469

[5] Pfortmueller CA, Dabrowski W, Wise R, van Regenmortel N, Malbrain MLNG. Fluid accumulation syndrome in sepsis and septic shock: pathophysiology, relevance and treatment-a comprehensive review. Ann Intensive Care. 2024;14:115. 39033219 10.1186/s13613-024-01336-9 PMC11264678

[6] Wollborn J, Hassenzahl LO, Reker D, Staehle HF, Omlor AM, Baar W, et al. Diagnosing capillary leak in critically ill patients: development of an innovative scoring instrument for non-invasive detection. Ann Intensive Care. 2021;11:175. 34910264 10.1186/s13613-021-00965-8 PMC8674404

[7] Starling EH. On the absorption of fluids from the connective tissue spaces. J Physiol. 1896;19:312–26. 16992325 10.1113/jphysiol.1896.sp000596 PMC1512609

[8] Levick JR, Michel CC. Microvascular fluid exchange and the revised starling principle. Cardiovasc Res. 2010;87:198–210. 20200043 10.1093/cvr/cvq062

[9] Executive Committee of the International Society of Lymphology. The diagnosis and treatment of peripheral lymphedema: 2020 Consensus Document of the International Society of Lymphology. Lymphology. 2020;53:3–19. 32521126

[10] Rabe E, Partsch H, Hafner J, Lattimer C, Mosti G, Neumann M, et al. Indications for medical compression stockings in venous and lymphatic disorders: an evidence-based consensus statement. Phlebology. 2018;33:163–84. 28549402 10.1177/0268355516689631 PMC5846867

[11] Ezzo J, Manheimer E, McNeely ML, Howell DM, Weiss R, Johansson KI, et al. Manual lymphatic drainage for lymphedema following breast cancer treatment. Cochrane Database Syst Rev. 2015;2015:CD003475. 25994425 10.1002/14651858.CD003475.pub2 PMC4966288

[12] Koul R, Dufan T, Russell C, Guenther W, Nugent Z, Sun X, et al. Efficacy of complete decongestive therapy and manual lymphatic drainage on treatment-related lymphedema in breast cancer. Int J Radiat Oncol Biol Phys. 2007;67:841–6. 17175115 10.1016/j.ijrobp.2006.09.024

[13] Dargent A, Large A, Soudry-Faure A, Doise J-M, Abdulmalak C, Jonval L, et al. Corporeal Compression at the Onset of Septic shock (COCOONs): a compression method to reduce fluid balance of septic shock patients. Sci Rep. 2019;9:11566. 31399609 10.1038/s41598-019-47939-2 PMC6689006

[14] Michard F, Alaya S, Zarka V, Bahloul M, Richard C, Teboul J-L. Global end-diastolic volume as an indicator of cardiac preload in patients with septic shock. Chest. 2003;124:1900–8. 14605066 10.1378/chest.124.5.1900

[15] Sakka SG, Rühl CC, Pfeiffer UJ, Beale R, McLuckie A, Reinhart K, et al. Assessment of cardiac preload and extravascular lung water by single transpulmonary thermodilution. Intensive Care Med. 2000;26:180–7. 10784306 10.1007/s001340050043

[16] Monnet X, Teboul J-L. Transpulmonary thermodilution: advantages and limits. Crit Care. 2017;21:147. 28625165 10.1186/s13054-017-1739-5 PMC5474867

[17] Committee E. The diagnosis and treatment of peripheral lymphedema: 2016 consensus document of the international society of lymphology. Lymphology. 2016;49:170–84. 29908550

[18] Belletti A, Lerose CC, Zangrillo A, Landoni G. Vasoactive-inotropic score: evolution, clinical utility, and pitfalls. J Cardiothorac Vasc Anesth. 2021;35:3067–77. 33069558 10.1053/j.jvca.2020.09.117

[19] Wichmann S, Barbateskovic M, Liang N, Itenov TS, Berthelsen RE, Lindschou J, et al. Loop diuretics in adult intensive care patients with fluid overload: a systematic review of randomised clinical trials with meta-analysis and trial sequential analysis. Ann Intensive Care. 2022;12:52. 35696008 10.1186/s13613-022-01024-6 PMC9192894

[20] Magder S. Volume and its relationship to cardiac output and venous return. Crit Care. 2016;20:271. 27613307 10.1186/s13054-016-1438-7 PMC5018186

[21] Hippensteel JA, Uchimido R, Tyler PD, Burke RC, Han X, Zhang F, et al. Intravenous fluid resuscitation is associated with septic endothelial glycocalyx degradation. Crit Care. 2019;23:259. 31337421 10.1186/s13054-019-2534-2 PMC6652002

[22] Sánchez M, Jiménez-Lendínez M, Cidoncha M, Asensio MJ, Herrerot E, Collado A, et al. Comparison of fluid compartments and fluid responsiveness in septic and non-septic patients. Anaesth Intensive Care. 2011;39:1022–9. 22165353 10.1177/0310057X1103900607

[23] Saravi B, Goebel U, Hassenzahl LO, Jung C, David S, Feldheiser A, et al. Capillary leak and endothelial permeability in critically ill patients: a current overview. Intensive Care Med Exp. 2023;11:96. 38117435 10.1186/s40635-023-00582-8 PMC10733291

[24] Bickel A, Shturman A, Sergeiev M, Ivry S, Eitan A, Atar S. Hemodynamic effect and safety of intermittent sequential pneumatic compression leg sleeves in patients with congestive heart failure. J Card Fail. 2014;20:739–46. 25038262 10.1016/j.cardfail.2014.07.004

[25] Magder S, De Varennes B. Clinical death and the measurement of stressed vascular volume. Crit Care Med. 1998;26:1061–4. 9635656 10.1097/00003246-199806000-00028

[26] Magder S. The use of Guyton’s approach to the control of cardiac output for clinical fluid management. Ann Intensive Care. 2024;14:105. 38963533 10.1186/s13613-024-01316-z PMC11224168

[27] Vincent J-L, Pinsky MR. We should avoid the term “fluid overload.” Crit Care. 2018;22:214. 30205826 10.1186/s13054-018-2141-7 PMC6134499

[28] Verbrugge FH, Dupont M, Steels P, Grieten L, Malbrain M, Tang WHW, et al. Abdominal contributions to cardiorenal dysfunction in congestive heart failure. J Am Coll Cardiol. 2013;62:485–95. 23747781 10.1016/j.jacc.2013.04.070

[29] Schefold JC, Filippatos G, Hasenfuss G, Anker SD, von Haehling S. Heart failure and kidney dysfunction: epidemiology, mechanisms and management. Nat Rev Nephrol. 2016;12:610–23. 27573728 10.1038/nrneph.2016.113

